# Morbidity and mortality in women with advanced ovarian cancer who underwent primary cytoreductive surgery compared to cytoreductive surgery for recurrent disease: a meta-analysis

**DOI:** 10.1515/pp-2019-0014

**Published:** 2019-06-28

**Authors:** Helena C. Bartels, Ailin C. Rogers, James Postle, Conor Shields, Jurgen Mulsow, John Conneely, Donal J. Brennan

**Affiliations:** Ireland East Hospital Gynaeoncology Group, Mater Misericordiae University, Dublin, Ireland; Department of Surgery, Mater Misericordiae University Hospital, Dublin, Ireland; UCD School of Medicine, National Maternity Hospital, Holles Street, Dublin, Ireland

**Keywords:** cytoreductive surgery, morbidity, ovarian cancer, recurrent malignancy

## Abstract

**Background:**

The primary treatment for advanced ovarian cancer is aggressive cytoreductive surgery (CRS), which is associated with considerable morbidity. The aim of this meta-analysis is to compare morbidity associated with primary CRS and secondary CRS for recurrent disease.

**Methods:**

A literature search was performed using Preferred Reporting Items for Systematic Reviews and Meta-Analyses (PRISMA) guidelines for publications reporting morbidity and mortality in patients undergoing CRS in primary and recurrent ovarian malignancy. Embase, Medline, Pubmed, Pubmed Central, clinicaltrials. gov and Cochrane databases were searched. Two independent reviewers applied inclusion and exclusion criteria to select included papers. A total of 215 citations were reviewed; 6 studies comprising 641 patients were selected for the analysis.

**Results:**

Results were reported as mean differences or pooled odds ratios (OR) with 95 % confidence intervals (95 % CI). The overall morbidity rate was 38.4 %, and this did not differ between the two groups (p=0.97). This did not change when only Clavien-Dindo grade 3 and 4 morbidities were accounted for (14 % primary CRS, 15 % recurrent, p=0.83). Compared to primary CRS, secondary CRS was associated with a similar operative time (mean 400 min, I2=79 %, p=0.45), rate of bowel resection (I2=75 %, p=0.37) and transfusion requirements (MD – 0.7 L, I2=76 %, p=0.45). The mortality rate in both groups was too low to allow for meaningful meta-analysis, with four deaths in the group undergoing primary cytoreductive surgery (1.0 %) and two deaths in the group with recurrent disease (0.9 %).

**Conclusions:**

In conclusion, secondary CRS for recurrent ovarian cancer is a safe and feasible option in carefully pre-selected patients with comparable morbidity to primary CRS.

## Introduction

Ovarian cancer is the ninth leading cause of cancer in women, but the fifth leading cause of all cancer-related deaths [1]. Disease often presents at an advanced stage, with 75 % of patients having metastatic disease at diagnosis – classically characterized by ascites, carcinomatosis and omental involvement [2]. This late presentation can be attributed in part to the lack of any specific symptoms until disease has spread, as well as the absence of a screening test for early detection. Where patients present with advanced disease and are suitable for surgical intervention, cytoreductive surgery (CRS), to achieve complete clearance of the abdominal cavity with no residual disease, is the gold standard of treatment and is associated with the most favorable survival outcomes [3].

CRS can be performed at the time of first diagnosis of ovarian cancer, when it is referred to as primary CRS, or for patients with recurrent disease as secondary cytoreduction. While primary CRS is accepted as a crucial step in the initial management of advanced ovarian cancer, the role of secondary CRS for recurrent disease is less well established. Radical cytoreduction, incorporating upper abdominal procedures such as diaphragmatic resection and splenectomy, has been shown to increase overall survival and progression free survival compared to more conservative CRS [4]. Previous studies have shown survival outcomes for recurrent disease are only achieved in carefully pre-selected patients with complete cytoreduction [5]. Hence careful patient selection in a multidisciplinary team setting is important to identify those who are likely to benefit from CRS, as it is associated with significant morbidity and mortality [3, 6] In order to confer any survival benefit, CRS should result in no macroscopic residual tumour with complete cytoreduction [7]. As previous studies defined optimal cytoreduciton with various criteria, meaningful comparative survival outcomes in secondary CRS were difficult [8]. To allow for a standardized method of reporting of residual disease the Gynecologic Oncology Group (GOG) has defined optimal cytoreduction as residual implants less than 1 cm [9]; however, complete cytoreduction to no visible disease confers an additional survival benefit and should be considered main objective of any CRS procedure.

Patients treated in specialist centers where a large volume of CRS are performed have shown improved survival and reduced morbidity [10]. The purpose of this meta-analysis is to review the morbidity and mortality associated with cytoreductive surgery in patients with primary and recurrent ovarian malignancy to assess if secondary CRS is comparable in terms of surgical complications.

## Sources

**Figure 1:**
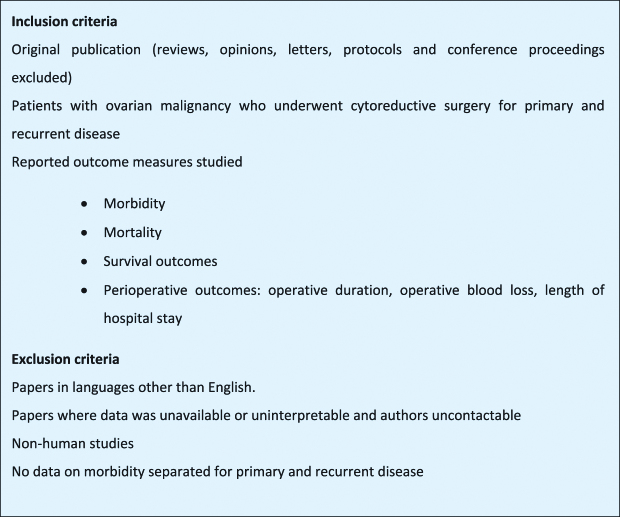
Study inclusion and exclusion criteria.

A systematic literature search was performed for all publications that reported on morbidity and mortality in patients undergoing cytoreductive surgery in primary and recurrent ovarian malignancy. Embase, Medline, PubMed, PubMed Central, clinicaltrials. gov and Cochrane databases were searched using a Boolean search algorithm for articles published up to January 2019. Moreover, the reference lists of the relevant literatures were also screened. Original studies documenting morbidity and mortality in patients undergoing primary and secondary CRS for ovarian malignancy, including both primary and recurrent disease, were included for meta-analysis. The overall search strategy was inclusive of alternative terms such as ovarian neoplasm, ovarian carcinoma, epithelial ovarian cancer, and synonyms for cytoreductive surgery (debulking surgery OR cytoreduction surgery OR primary cytoreduction OR secondary cytoreduction). Publications were evaluated dependent on predefined inclusion and exclusion criteria (Figure 1). All search results were combined in a reference manager database (Endnote™, Version X7, Thompson Reuters, New York, USA) and duplicates were removed by hand. Reference lists of included studies were screened for additional relevant studies.

## Study selection

The inclusion and exclusion criteria were applied to retrieve citations by two independent reviewers and the abstracts were reviewed to select full papers for data analysis. Full text studies were further evaluated, and exclusion criteria were applied to identify final papers for inclusion. Additional discrepancies were agreed by consensus. For each study, data on baseline characteristics (author institution, country, study period, total number of patients, surgical procedures performed, follow-up period and study methodology) were extracted. Periprocedural outcomes included stage at diagnosis, histological subtype, cytoreductive score, estimated blood loss (EBL, expressed in litres [L]), units of red cells transfused, length of stay (LOS), morbidity and mortality. Morbidities were recorded qualitatively and quantitatively.

The meta-analysis was conducted in accordance with PRISMA guidelines. Study methodological quality was assessed by applying the MINORS criteria for observational studies [11, 12] Authors were contacted if data were not available or uninterpretable, with additional morbidity data provided by two authors [13, 14].

Analyses were performed using RevMan software (Review Manager, version 5.3; The Nordic Cochrane Centre, The Cochrane Collaboration, Copenhagen, Denmark). Continuous data were presented as mean±standard deviation and statistical significance was interpreted using the two tailed t-test. When median and range were presented, methods described by Hozo and colleagues were followed to derive mean and standard deviation [15]. Association of categorical variables (differences for dichotomous pre-existing variables between groups) was assessed using chi-square (χ^2^) test or Fisher’s test where appropriate. Cochran’s Q-test was used to calculate the I^2^ statistic in order to objectively measure heterogeneity for each of the outcome measures; an I^2^ value greater than 50 % was taken to denote significant heterogeneity between studies. A fixed-effects model was performed for each variable, or where there was appreciable heterogeneity (I^2^>50 %) a random-effects model was used for meta-analysis. For continuous variables, the weighted mean differences (MD) are presented with 95 % confidence intervals (CI). For categorical variables Mantel–Haenszel odds ratios (ORs) were calculated and described with 95 % CI. Corresponding funnel plots of log standard error as a function of effect size were used to examine the effect of publication bias visually. Subgroup analysis of those studies utilizing Hyperthermic IntraPeritoneal Chemotherapy (HIPEC) in addition to CRS was performed, and sensitivity analysis omitted studies if they scored poorly for methodological quality (MINORS score <15). p-Values were two tailed, differences of <0.05 were deemed to be significant.

## Results

**Figure 2:**
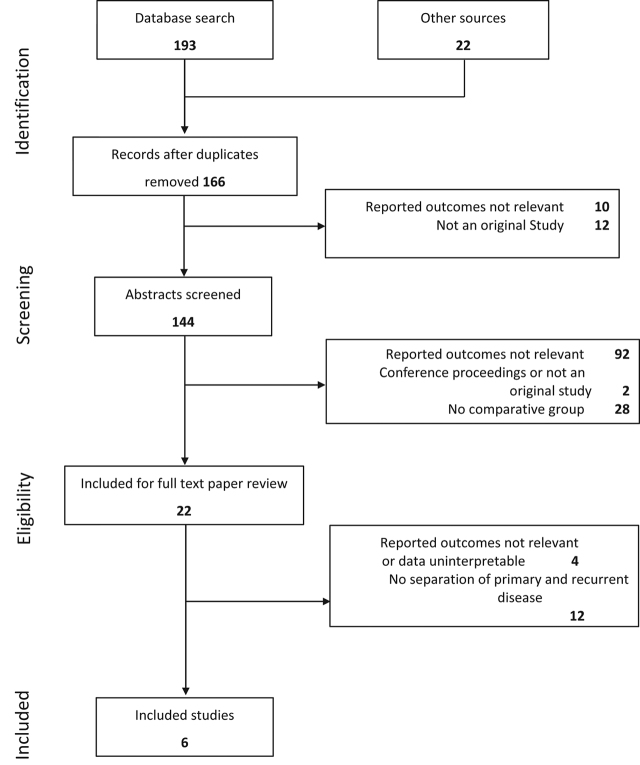
Flow diagram of systematic review and meta-analysis process.

A comprehensive search of databases resulted in a total of 215 papers, of which 166 remained for review after removal of duplicate papers. Following review of titles, abstracts, 22 full text papers remained for analysis. Six studies were eventually included after meeting the study inclusion criteria (Figure 2). There were 641 patients in total, of whom 64.9 % had primary disease and the remainder had recurrence. Study sizes ranged from 33 to 222 patients. Five studies were retrospective and one a prospective phase 2 study (Table 1). Of the six papers included, three were from Spain, one from Italy, one from Germany and one from Korea. All papers were published between 2006 and 2017, with patients included from 1996 to 2014.

**Table 1:** Overview of included studies.

Author	Year	Country	Study period	Study type	Total n	Primary ovarian cancer (%)	Recurrent ovarian cancer (%)	Intervention		MINORS score
								Primary ovarian cancer	Recurrent ovarian cancer	
Park [16]	2006	Korea	2001–2005	Retrospective cohort study	60	46 (76.7)	14 (23.3)	PRCS+HIPEC	SCRS+HIPEC	15
Rufián [17]	2006	Spain	1997–2004	Retrospective cohort study	33	19 (57.6)	14 (42.4)	PRCS+HIPEC	SCRS+HIPEC	15
Di Giorgio A [18]	2008	Italy	2000–2007	Non randomized phase 2 study, open, prospective	47	22 (46.8)	25 (53.2)	PRCS HIPEC: Cisplatin+doxorubicin, paclitaxel	SCRS HIPEC: Cisplatin+doxorubicin, paclitaxel	17
Woelber [19]	2010	Germany	1996–2004	Retrospective cohort study	222	174 (78.3)	48 (21.7)	Radical CRS	Radical CRS	16
Muñoz-Casares FC [20]	2016	Spain	1996–2012	Retrospective cohort study	218	124 (56.6)	94 (44.4)	PCRS+4–8cycles of carboplatin+paclitaxel neoadjuvant chemotherapy	SCRS 4–8cycles of carboplatin+paclitaxel neoadjuvant chemotherapy	16
Manzendo [21]	2017	Spain	2007–2014	Retrospective cohort study	61	31 (51)	30 (49)	PRCS+HIPEC	SCRS+HIPEC	15

PCRS, primary cytoreductive surgery; SCRS, secondary cytoreductive surgery; HIPEC, Hyperthermic IntraPEritoneal Chemotherapy.

Four studies described cytoreductive surgery with concomitant HIPEC, and the others without HIPEC (Table 1). All patients were at least FIGO stage 3, except 12 in the group with recurrent disease [12, 13]. The majority of patients (69.7 %) had high grade serous adenocarcinoma, with the remainder having a typically representative variety of histological subtypes (Table 2).

**Table 2:** Histological subtype [12, 13, 15, 22, 23].

Histological subtype	Primary ovarian cancer (n=385)	Recurrent ovarian cancer (n=195)	p-Value
Serous	286	118	0.164
Endometrioid	27	20	0.263
Mucinous	13	18	0.016
Undifferentiated	23	20	0.097
Clear cell/Carcinosarcoma	6	4	0.739
Unknown	30	15	

Tumour burden was estimated by the Peritoneal Cancer Index (PCI) in three studies [16, 18, 19]. Muñoz-Casares reported a higher PCI in those with primary disease, with 26 % scoring higher than 20 compared to 17 % (p=0.003) in the recurrent group, while Manzendo and Di Giorgio found no difference in PCI score between the two groups.

The extent of cytoreduction was described in four studies [3, 17, 18] with the extent of peritonectomy procedures similarly distributed among the two groups.

### Meta-analysis outcomes

#### Perioperative outcomes

**Figure 3:**
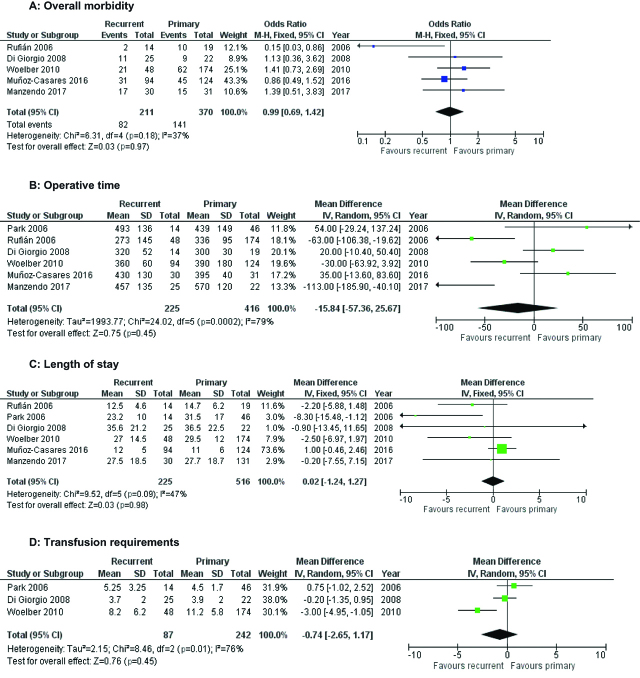
Forest plots of morbidity outcomes.

All studies reported the operative duration, with a mean overall operative time of 400 min (Primary CRS 356.5± 154.9, secondary CRS 356.5±72). There were no differences between surgical operating times on primary vs. recurrent disease at random effects meta-analysis (I^2^=79 %, p=0.45 Figure 3). All studies also described cases of intestinal resection, with no significant preponderance toward resection in primary vs. recurrent cases on random effects meta-analysis (I^2^=75 %, p=0.37, Table 3). Only two studies reported estimated intraoperative blood loss, and Park found no difference between the two groups, while Di Georgio found more blood loss in the group undergoing surgery for primary disease (mean 2.1 L vs. 1.5 L, p=0.01). Another study assessed the number of units of red cells prescribed [13], and a meta-analysis of the three found no difference in units transfused between groups at random effects meta-analysis (MD − 0.7 L, I^2^=76 %, p=0.45, Figure 3D)).

**Table 3:** Qualitative representation of morbidities [15, 22, 23].

Postoperative complication	Primary cancer (n=239)	Recurrent cancer (n=76)	p-Value
Leakage of Anastomotic Site	1	0	0.573
Rectovaginal Fistula	1	1	0.427
Pancreatic Juice Leakage	1	1	0.427
Ileus	6	1	1.000
Febrile Morbidity	11	6	0.384
Pleural Effusion	15	3	0.580
Atelectasis	4	2	0.635
Bile Leakage	0	1	0.243
Wound Dehiscence	2	5	0.012
Hemoperitoneum	0	1	0.243
Urinary Tract Infection	4	4	0.107
Acute Renal Failure	1	1	0.427
Reoperation	21	3	0.314
Pneumonia	2	0	0.566
DVT/PE	11	2	0.740
Bowel Perforation	4	4	0.107

DVT, deep venous thrombosis; PE, pulmonary embolism.

#### Postoperative morbidity, mortality and length of stay

Perioperative morbidity was detailed in five studies [13, 14, 15, 22, 23]. The overall morbidity rate was 38.4 % for all Clavien-Dindo grades 1–4, and this did not differ between the two groups (36 % in primary CRS, 40 % in recurrent, p=0.97 Figure 3A). This did not change when only Clavien-Dindo grade 3 and 4 morbidities were accounted for (14 % primary CRS, 15 % recurrent, p=0.83). The reintervention rate was not insignificant, with 8.4 % (49 patients) requiring return to theatre. Reasons for reintervention were only outlined in two studies [12, 23] and included anastomotic leak, hemorrhage, other visceral perforation, rectovaginal fistula or intraabdominal abscess. Although all studies commented on postoperative mortality, there were too few deaths in either group to allow meaningful meta-analysis, with four deaths in the group undergoing primary cytoreductive surgery (1.0 %) and two deaths in the group with recurrent disease (0.9 %). There was no significant difference in length of stay between the two groups (p=0.98), with an overall reported mean length of stay of 23.3±10.0 days (Figure 3C).

#### Oncological outcomes

The rate of complete (R0) resection was 69.4 %, as outlined in five studies [12, 14, 15, 22, 24] with no significant difference between the two groups (p=0.46). R1 resection was achieved in 21.6 % of patients and R2 in 9 %, with no difference between the two groups (p=0.23 and p=1.0, respectively). Two studies reported lymph node status, and again there were no differences between groups [15, 23].

Muñoz-Casares [23] reported those who were disease-free at 48 months, with rates of 49.4 % in those women undergoing primary cytoreductive surgery vs. 38.8 % of those having recurrent surgery, although the low numbers in this study fail to achieve statistical significance (p=0.20). Two other studies reported median disease-free survival after CRS for primary or recurrent ovarian cancer, with no significant difference between groups in Woelber’s [13] study (21 vs. 22 months), and 25.5 vs. 15.5 months in Di Georgio’s [22] cohort. There were also no significant differences in 5-year survival between the two groups (47.1 vs. 48.9 %, I^2^=0 %, p=0.74).

Funnel plots revealed substantial heterogeneity among all perioperative outcomes but not postoperative or oncological outcomes. A subgroup analysis was performed to consider only those studies which used HIPEC as an operative strategy with CRS. Exclusion of studies not utilizing HIPEC still failed to demonstrate any significant difference in any outcome for those undergoing CRS for primary vs. recurrent ovarian cancer. No study scored less than 15 when MINORS criteria were applied, and thus a sensitivity analysis was not required.

## Discussion

This meta-analysis demonstrates that secondary CRS can be performed with a similar morbidity and mortality as primary CRS in advanced ovarian cancer. While the overall morbidity and reoperation rates were not insignificant, the potential gains in survival associated with surgery validate CRS in recurrent disease as a beneficial option in carefully selected patients.

Patient selection is crucial as previous studies have shown improved survival in recurrent disease only in patients with pre-defined criteria; the AGO DESKTOP 1 study, which was a retrospective analysis of case records, showed improved survival only in those patients with a good performance status, complete resection at first surgery and <500 mL ascites – which was developed as the AGO score [4]. The AGO score was validated in DESKTOP 2, a prospective study that demonstrated a 75 % complete resection rate women who met the pre-specified criteria. Secondary CRS was associated with a considerable complication rate in patients with 33 % having at least one complication and 11 % requiring reoperation [22]. Final results from the prospective randomized DESKTOP 3 trial comparing secondary CRS and chemotherapy to chemotherapy alone in women with a positive AGO score are awaited, however preliminary results suggest a progression free survival benefit if complete gross resection can be achieved [13].

However, another prospective randomized control trial, GOG213 [23] found no benefit in overall survival or progression free survival in patients with recurrent disease who underwent secondary CRS compared to chemotherapy only when less stringent selection criteria were used. Hence a comparable morbidity rate at secondary CRS is an important finding to further justify surgical intervention in recurrent disease with questionable improvements in survival, particularly as the final results and overall survival data from DESKTOP-III are awaited. The primary aim of this meta-analysis was to compare morbidity in patients undergoing primary and secondary CRS, however we also have found no difference in disease free survival or 5 year survival between the two groups.

Compared to morbidity in CRS performed for non-gynecological malignancies, where a peri-operative complication rate of 27–56 % is reported in a number of studies [14], the morbidity rates reported here are favorable. Given the questionable benefit of secondary CRS for ovarian cancer, it is likely that younger patients with an excellent performance status will be offered surgery for recurrent disease, which may translate into lower peri-operative complication rates.

The main strength of this meta-analysis is that, to our knowledge, this is the first review to compare morbidity in patients with ovarian cancer undergoing CRS in primary and recurrent disease, and hence provides important evidence that morbidity is not increased at secondary CRS in carefully selected patients. As 80–90 % of patients with ovarian cancer will eventually develop recurrent disease [3], the morbidity of any surgical intervention should be clearly documented and understood.

This meta-analysis has several limitations. As with any meta-analysis, the conclusions that can be drawn are subject to the limitations of the included studies. Notably, all except one of the included studies are retrospective and performed in single institutions, hence the risk of bias is high. The included studies used different institution specific surgical and chemotherapy regimens which will also have an effect on the reported results. Furthermore, although Chi *et al.* produced a set of guidelines to aid in patient selection for secondary CRS [24], institutions will ultimately determine if a patient is deemed suitable for secondary CRS leading to significant selection bias.

In conclusion, secondary CRS for recurrent ovarian cancer is a reasonable approach in carefully pre-selected patients with comparable morbidity to primary CRS.
